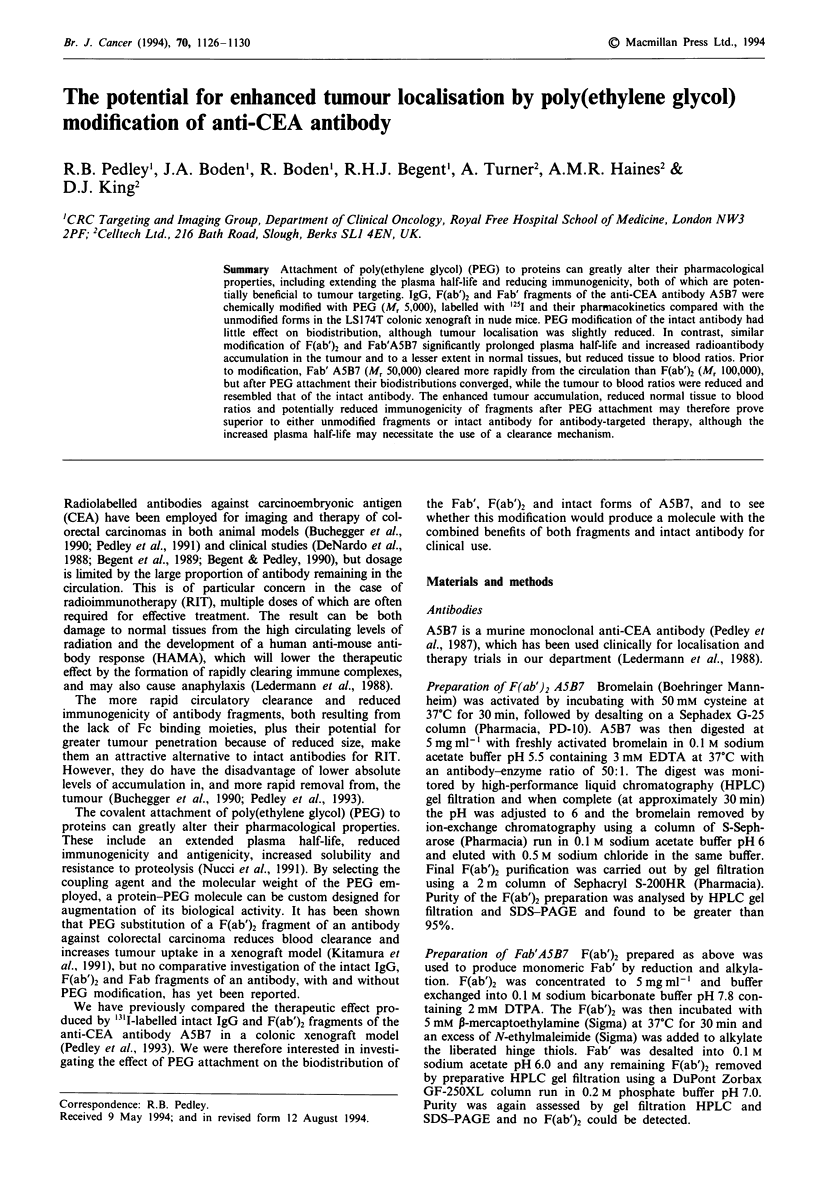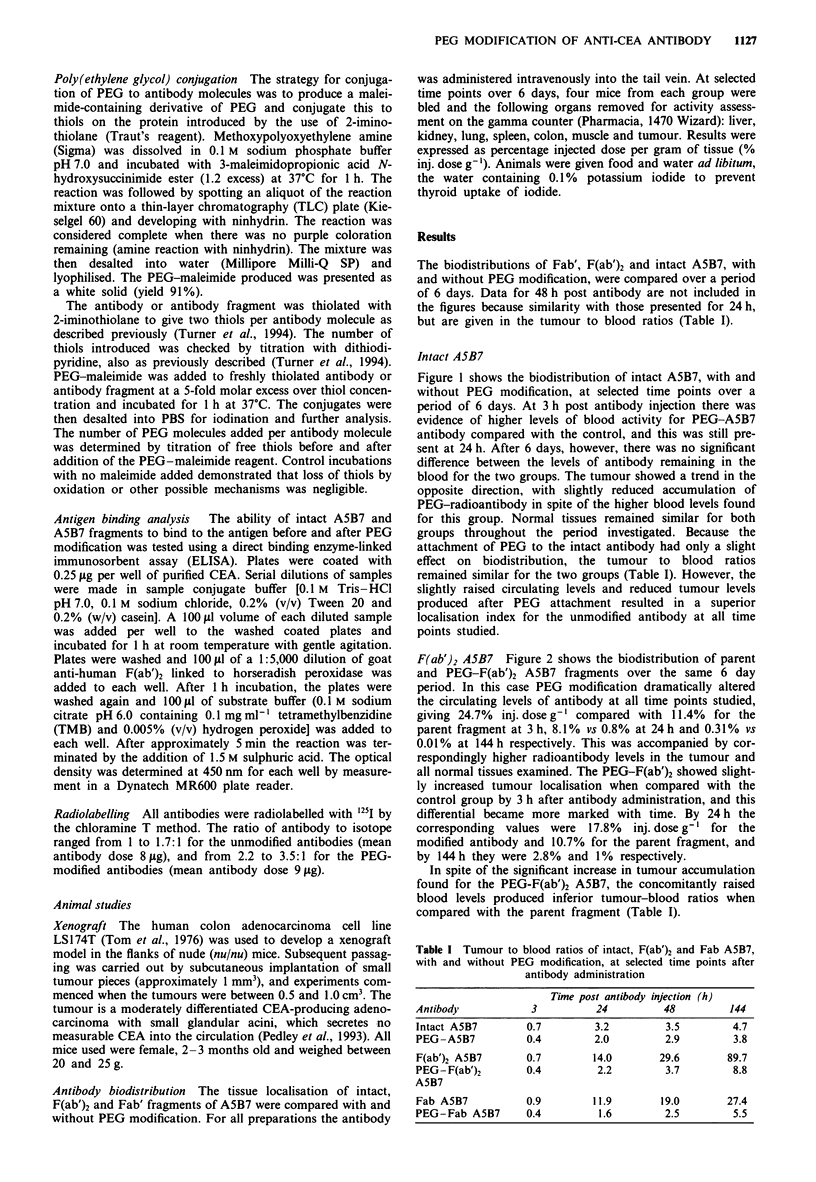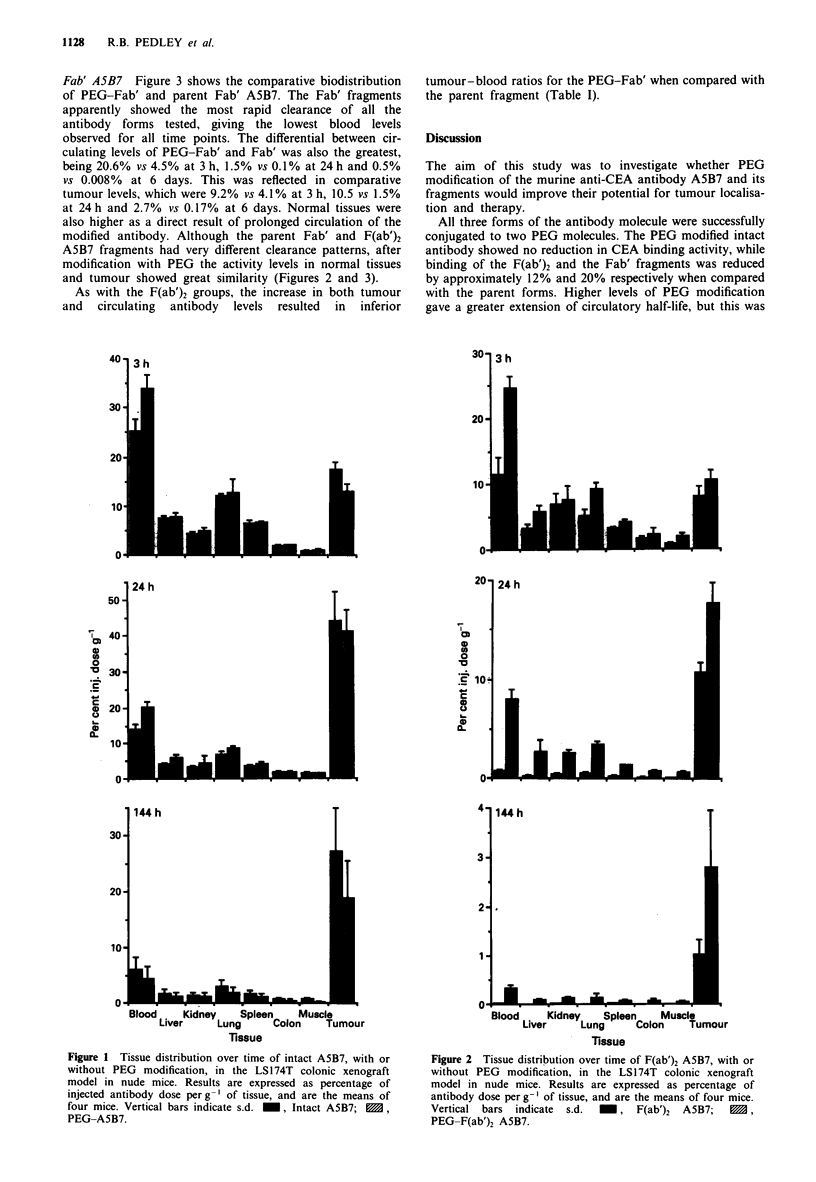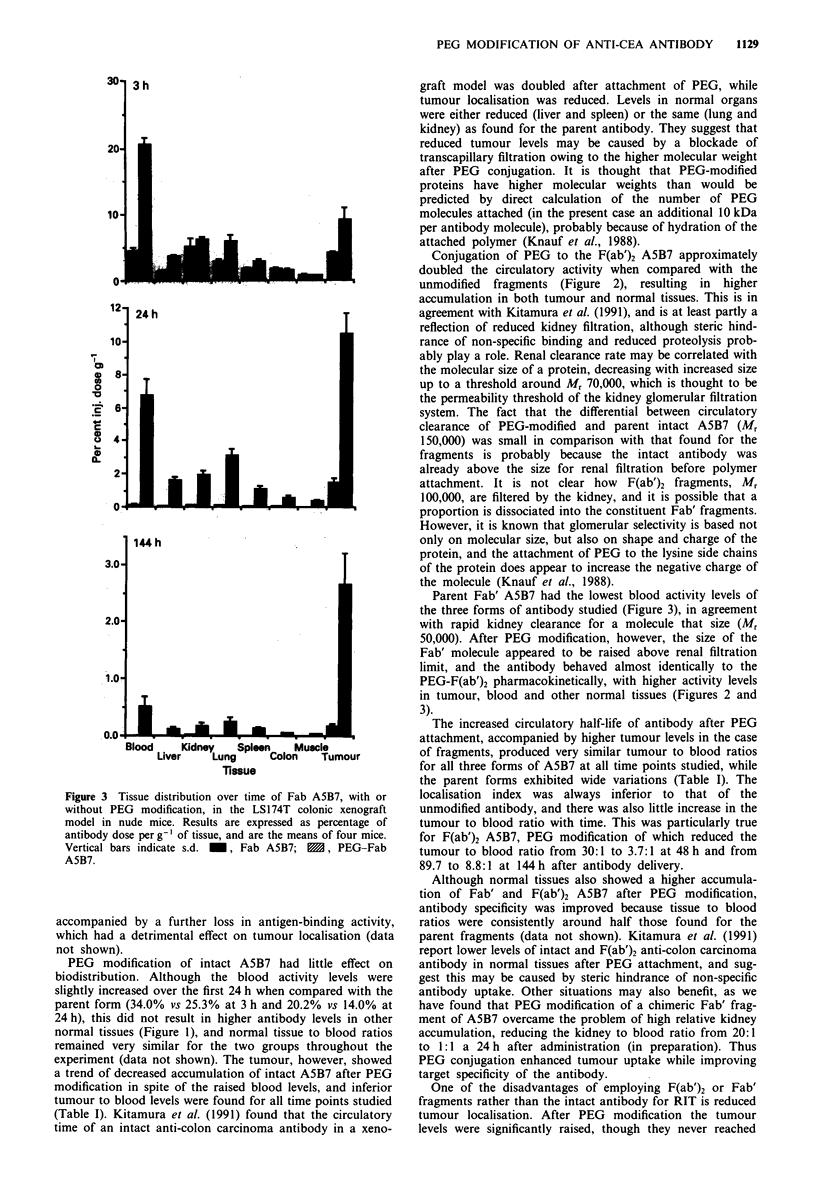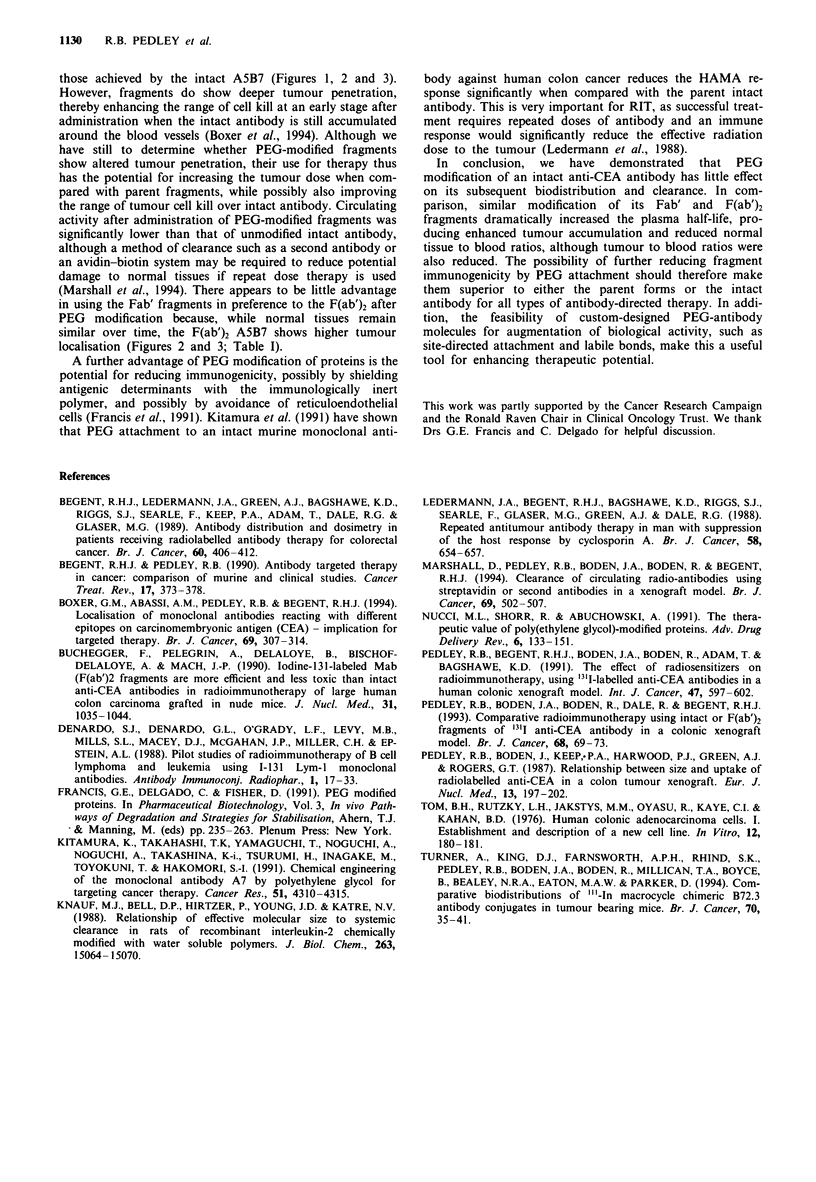# The potential for enhanced tumour localisation by poly(ethylene glycol) modification of anti-CEA antibody.

**DOI:** 10.1038/bjc.1994.459

**Published:** 1994-12

**Authors:** R. B. Pedley, J. A. Boden, R. Boden, R. H. Begent, A. Turner, A. M. Haines, D. J. King

**Affiliations:** Department of Clinical Oncology, Royal Free Hospital School of Medicine, London, U.K.

## Abstract

Attachment of poly(ethylene glycol) (PEG) to proteins can greatly alter their pharmacological properties, including extending the plasma half-life and reducing immunogenicity, both of which are potentially beneficial to tumour targeting. IgG, F(ab')2 and Fab' fragments of the anti-CEA antibody A5B7 were chemically modified with PEG (M(r) 5,000), labelled with 125I and their pharmacokinetics compared with the unmodified forms in the LS174T colonic xenograft in nude mice. PEG modification of the intact antibody had little effect on biodistribution, although tumour localisation was slightly reduced. In contrast, similar modification of F(ab')2 and Fab'A5B7 significantly prolonged plasma half-life and increased radioantibody accumulation in the tumour and to a lesser extent in normal tissues, but reduced tissue to blood ratios. Prior to modification, Fab' A5B7 (M(r) 50,000) cleared more rapidly from the circulation than F(ab')2 (M(r) 100,000), but after PEG attachment their biodistributions converged, while the tumour to blood ratios were reduced and resembled that of the intact antibody. The enhanced tumour accumulation, reduced normal tissue to blood ratios and potentially reduced immunogenicity of fragments after PEG attachment may therefore prove superior to either unmodified fragments or intact antibody for antibody-targeted therapy, although the increased plasma half-life may necessitate the use of a clearance mechanism.


Br. J. Cancer (1994), 70, 1126–1130 © Macmillan Press Ltd., 1994

### The potential for enhanced tumour localisation by poly(ethylene glycol) modification of anti-CEA antibody

R.B. Pedley[1], J.A. Boden[1], R. Boden[1], R.H.J. Begent[1], A. Turner[2], A.M.R. Haines[2] & D.J. King[2]

[1]*CRC Targeting and Imaging Group, Department of Clinical Oncology, Royal Free Hospital School of Medicine, London NW3 2PF;* [2]*Celltech Ltd., 216 Bath Road, Slough, Berks SL1 4EN, UK.*

**Summary** Attachment of poly(ethylene glycol) (PEG) to proteins can greatly alter their pharmacological properties, including extending the plasma half-life and reducing immunogenicity, both of which are potentially beneficial to tumour targeting. IgG, $F(ab')_2$ and Fab' fragments of the anti-CEA antibody A5B7 were chemically modified with PEG ($M_r$ 5,000), labelled with $^{125}I$ and their pharmacokinetics compared with the unmodified forms in the LS174T colonic xenograft in nude mice. PEG modification of the intact antibody had little effect on biodistribution, although tumour localisation was slightly reduced. In contrast, similar modification of $F(ab')_2$ and Fab'A5B7 significantly prolonged plasma half-life and increased radioantibody accumulation in the tumour and to a lesser extent in normal tissues, but reduced tissue to blood ratios. Prior to modification, Fab' A5B7 ($M_r$ 50,000) cleared more rapidly from the circulation than $F(ab')_2$ ($M_r$ 100,000), but after PEG attachment their biodistributions converged, while the tumour to blood ratios were reduced and resembled that of the intact antibody. The enhanced tumour accumulation, reduced normal tissue to blood ratios and potentially reduced immunogenicity of fragments after PEG attachment may therefore prove superior to either unmodified fragments or intact antibody for antibody-targeted therapy, although the increased plasma half-life may necessitate the use of a clearance mechanism.

Radiolabelled antibodies against carcinoembryonic antigen (CEA) have been employed for imaging and therapy of colorectal carcinomas in both animal models (Buchegger *et al.*, 1990; Pedley *et al.*, 1991) and clinical studies (DeNardo *et al.*, 1988; Begent *et al.*, 1989; Begent & Pedley, 1990), but dosage is limited by the large proportion of antibody remaining in the circulation. This is of particular concern in the case of radioimmunotherapy (RIT), multiple doses of which are often required for effective treatment. The result can be both damage to normal tissues from the high circulating levels of radiation and the development of a human anti-mouse antibody response (HAMA), which will lower the therapeutic effect by the formation of rapidly clearing immune complexes, and may also cause anaphylaxis (Ledermann *et al.*, 1988).

The more rapid circulatory clearance and reduced immunogenicity of antibody fragments, both resulting from the lack of Fc binding moieties, plus their potential for greater tumour penetration because of reduced size, make them an attractive alternative to intact antibodies for RIT. However, they do have the disadvantage of lower absolute levels of accumulation in, and more rapid removal from, the tumour (Buchegger *et al.*, 1990; Pedley *et al.*, 1993).

The covalent attachment of poly(ethylene glycol) (PEG) to proteins can greatly alter their pharmacological properties. These include an extended plasma half-life, reduced immunogenicity and antigenicity, increased solubility and resistance to proteolysis (Nucci *et al.*, 1991). By selecting the coupling agent and the molecular weight of the PEG employed, a protein–PEG molecule can be custom designed for augmentation of its biological activity. It has been shown that PEG substitution of a $F(ab')_2$ fragment of an antibody against colorectal carcinoma reduces blood clearance and increases tumour uptake in a xenograft model (Kitamura *et al.*, 1991), but no comparative investigation of the intact IgG, $F(ab')_2$ and Fab fragments of an antibody, with and without PEG modification, has yet been reported.

We have previously compared the therapeutic effect produced by $^{131}I$-labelled intact IgG and $F(ab')_2$ fragments of the anti-CEA antibody A5B7 in a colonic xenograft model (Pedley *et al.*, 1993). We were therefore interested in investigating the effect of PEG attachment on the biodistribution of the Fab', $F(ab')_2$ and intact forms of A5B7, and to see whether this modification would produce a molecule with the combined benefits of both fragments and intact antibody for clinical use.

Correspondence: R.B. Pedley.
Received 9 May 1994; and in revised form 12 August 1994.

### Materials and methods

### *Antibodies*

A5B7 is a murine monoclonal anti-CEA antibody (Pedley *et al.*, 1987), which has been used clinically for localisation and therapy trials in our department (Ledermann *et al.*, 1988).

*Preparation of $F(ab')_2$ A5B7* Bromelain (Boehringer Mannheim) was activated by incubating with 50 mM cysteine at 37°C for 30 min, followed by desalting on a Sephadex G-25 column (Pharmacia, PD-10). A5B7 was then digested at 5 mg ml$^{-1}$ with freshly activated bromelain in 0.1 M sodium acetate buffer pH 5.5 containing 3 mM EDTA at 37°C with an antibody–enzyme ratio of 50:1. The digest was monitored by high-performance liquid chromatography (HPLC) gel filtration and when complete (at approximately 30 min) the pH was adjusted to 6 and the bromelain removed by ion-exchange chromatography using a column of S-Sepharose (Pharmacia) run in 0.1 M sodium acetate buffer pH 6 and eluted with 0.5 M sodium chloride in the same buffer. Final $F(ab')_2$ purification was carried out by gel filtration using a 2 m column of Sephacryl S-200HR (Pharmacia). Purity of the $F(ab')_2$ preparation was analysed by HPLC gel filtration and SDS–PAGE and found to be greater than 95%.

*Preparation of Fab'A5B7* $F(ab')_2$ prepared as above was used to produce monomeric Fab' by reduction and alkylation. $F(ab')_2$ was concentrated to 5 mg ml$^{-1}$ and buffer exchanged into 0.1 M sodium bicarbonate buffer pH 7.8 containing 2 mM DTPA. The $F(ab')_2$ was then incubated with 5 mM β-mercaptoethylamine (Sigma) at 37°C for 30 min and an excess of *N*-ethylmaleimide (Sigma) was added to alkylate the liberated hinge thiols. Fab' was desalted into 0.1 M sodium acetate pH 6.0 and any remaining $F(ab')_2$ removed by preparative HPLC gel filtration using a DuPont Zorbax GF-250XL column run in 0.2 M phosphate buffer pH 7.0. Purity was again assessed by gel filtration HPLC and SDS–PAGE and no $F(ab')_2$ could be detected.

*Poly(ethylene glycol) conjugation* The strategy for conjugation of PEG to antibody molecules was to produce a maleimide-containing derivative of PEG and conjugate this to thiols on the protein introduced by the use of 2-iminothiolane (Traut's reagent). Methoxypolyoxyethylene amine (Sigma) was dissolved in 0.1 M sodium phosphate buffer pH 7.0 and incubated with 3-maleimidopropionic acid *N*-hydroxysuccinimide ester (1.2 excess) at 37°C for 1 h. The reaction was followed by spotting an aliquot of the reaction mixture onto a thin-layer chromatography (TLC) plate (Kieselgel 60) and developing with ninhydrin. The reaction was considered complete when there was no purple coloration remaining (amine reaction with ninhydrin). The mixture was then desalted into water (Millipore Milli-Q SP) and lyophilised. The PEG–maleimide produced was presented as a white solid (yield 91%).

The antibody or antibody fragment was thiolated with 2-iminothiolane to give two thiols per antibody molecule as described previously (Turner *et al.*, 1994). The number of thiols introduced was checked by titration with dithiodipyridine, also as previously described (Turner *et al.*, 1994). PEG–maleimide was added to freshly thiolated antibody or antibody fragment at a 5-fold molar excess over thiol concentration and incubated for 1 h at 37°C. The conjugates were then desalted into PBS for iodination and further analysis. The number of PEG molecules added per antibody molecule was determined by titration of free thiols before and after addition of the PEG-maleimide reagent. Control incubations with no maleimide added demonstrated that loss of thiols by oxidation or other possible mechanisms was negligible.

*Antigen binding analysis* The ability of intact A5B7 and A5B7 fragments to bind to the antigen before and after PEG modification was tested using a direct binding enzyme-linked immunosorbent assay (ELISA). Plates were coated with 0.25 µg per well of purified CEA. Serial dilutions of samples were made in sample conjugate buffer [0.1 M Tris-HCl pH 7.0, 0.1 M sodium chloride, 0.2% (v/v) Tween 20 and 0.2% (w/v) casein]. A 100 µl volume of each diluted sample was added per well to the washed coated plates and incubated for 1 h at room temperature with gentle agitation. Plates were washed and 100 µl of a 1:5,000 dilution of goat anti-human $F(ab')_2$ linked to horseradish peroxidase was added to each well. After 1 h incubation, the plates were washed again and 100 µl of substrate buffer (0.1 M sodium citrate pH 6.0 containing 0.1 mg ml$^{-1}$ tetramethylbenzidine (TMB) and 0.005% (v/v) hydrogen peroxide] was added to each well. After approximately 5 min the reaction was terminated by the addition of 1.5 M sulphuric acid. The optical density was determined at 450 nm for each well by measurement in a Dynatech MR600 plate reader.

*Radiolabelling* All antibodies were radiolabelled with $^{125}I$ by the chloramine T method. The ratio of antibody to isotope ranged from 1 to 1.7:1 for the unmodified antibodies (mean antibody dose 8 µg), and from 2.2 to 3.5:1 for the PEG-modified antibodies (mean antibody dose 9 µg).

### *Animal studies*

*Xenograft* The human colon adenocarcinoma cell line LS174T (Tom *et al.*, 1976) was used to develop a xenograft model in the flanks of nude (*nu/nu*) mice. Subsequent passaging was carried out by subcutaneous implantation of small tumour pieces (approximately 1 mm$^3$), and experiments commenced when the tumours were between 0.5 and 1.0 cm$^3$. The tumour is a moderately differentiated CEA-producing adenocarcinoma with small glandular acini, which secretes no measurable CEA into the circulation (Pedley *et al.*, 1993). All mice used were female, 2-3 months old and weighed between 20 and 25 g.

*Antibody biodistribution* The tissue localisation of intact, $F(ab')_2$ and Fab' fragments of A5B7 were compared with and without PEG modification. For all preparations the antibody was administered intravenously into the tail vein. At selected time points over 6 days, four mice from each group were bled and the following organs removed for activity assessment on the gamma counter (Pharmacia, 1470 Wizard): liver, kidney, lung, spleen, colon, muscle and tumour. Results were expressed as percentage injected dose per gram of tissue (% inj. dose g$^{-1}$). Animals were given food and water *ad libitum*, the water containing 0.1% potassium iodide to prevent thyroid uptake of iodide.

### Results

The biodistributions of Fab', $F(ab')_2$ and intact A5B7, with and without PEG modification, were compared over a period of 6 days. Data for 48 h post antibody are not included in the figures because similarity with those presented for 24 h, but are given in the tumour to blood ratios (Table I).

### *Intact A5B7*

Figure 1 shows the biodistribution of intact A5B7, with and without PEG modification, at selected time points over a period of 6 days. At 3 h post antibody injection there was evidence of higher levels of blood activity for PEG–A5B7 antibody compared with the control, and this was still present at 24 h. After 6 days, however, there was no significant difference between the levels of antibody remaining in the blood for the two groups. The tumour showed a trend in the opposite direction, with slightly reduced accumulation of PEG–radioantibody in spite of the higher blood levels found for this group. Normal tissues remained similar for both groups throughout the period investigated. Because the attachment of PEG to the intact antibody had only a slight effect on biodistribution, the tumour to blood ratios remained similar for the two groups (Table I). However, the slightly raised circulating levels and reduced tumour levels produced after PEG attachment resulted in a superior localisation index for the unmodified antibody at all time points studied.

*$F(ab')_2$ A5B7* Figure 2 shows the biodistribution of parent and PEG–$F(ab')_2$ A5B7 fragments over the same 6 day period. In this case PEG modification dramatically altered the circulating levels of antibody at all time points studied, giving 24.7% inj. dose g$^{-1}$ compared with 11.4% for the parent fragment at 3 h, 8.1% *vs* 0.8% at 24 h and 0.31% *vs* 0.01% at 144 h respectively. This was accompanied by correspondingly higher radioantibody levels in the tumour and all normal tissues examined. The PEG–$F(ab')_2$ showed slightly increased tumour localisation when compared with the control group by 3 h after antibody administration, and this differential became more marked with time. By 24 h the corresponding values were 17.8% inj. dose g$^{-1}$ for the modified antibody and 10.7% for the parent fragment, and by 144 h they were 2.8% and 1% respectively.

In spite of the significant increase in tumour accumulation found for the PEG-$F(ab')_2$ A5B7, the concomitantly raised blood levels produced inferior tumour–blood ratios when compared with the parent fragment (Table I).

**Table I** Tumour to blood ratios of intact, $F(ab')_2$ and Fab A5B7, with and without PEG modification, at selected time points after antibody administration

| | *Time post antibody injection (h)* | | | |
|---|---|---|---|---|
| *Antibody* | *3* | *24* | *48* | *144* |
| Intact A5B7 | 0.7 | 3.2 | 3.5 | 4.7 |
| PEG-A5B7 | 0.4 | 2.0 | 2.9 | 3.8 |
| $F(ab')_2$ A5B7 | 0.7 | 14.0 | 29.6 | 89.7 |
| PEG-$F(ab')_2$ A5B7 | 0.4 | 2.2 | 3.7 | 8.8 |
| Fab A5B7 | 0.9 | 11.9 | 19.0 | 27.4 |
| PEG-Fab A5B7 | 0.4 | 1.6 | 2.5 | 5.5 |

*Fab' A5B7* Figure 3 shows the comparative biodistribution of PEG–Fab' and parent Fab' A5B7. The Fab' fragments apparently showed the most rapid clearance of all the antibody forms tested, giving the lowest blood levels observed for all time points. The differential between circulating levels of PEG–Fab' and Fab' was also the greatest, being 20.6% *vs* 4.5% at 3 h, 1.5% *vs* 0.1% at 24 h and 0.5% *vs* 0.008% at 6 days. This was reflected in comparative tumour levels, which were 9.2% *vs* 4.1% at 3 h, 10.5 *vs* 1.5% at 24 h and 2.7% *vs* 0.17% at 6 days. Normal tissues were also higher as a direct result of prolonged circulation of the modified antibody. Although the parent Fab' and $F(ab')_2$ A5B7 fragments had very different clearance patterns, after modification with PEG the activity levels in normal tissues and tumour showed great similarity (Figures 2 and 3).

As with the $F(ab')_2$ groups, the increase in both tumour and circulating antibody levels resulted in inferior tumour–blood ratios for the PEG–Fab' when compared with the parent fragment (Table I).

### Discussion

The aim of this study was to investigate whether PEG modification of the murine anti-CEA antibody A5B7 and its fragments would improve their potential for tumour localisation and therapy.

All three forms of the antibody molecule were successfully conjugated to two PEG molecules. The PEG modified intact antibody showed no reduction in CEA binding activity, while binding of the $F(ab')_2$ and the Fab' fragments was reduced by approximately 12% and 20% respectively when compared with the parent forms. Higher levels of PEG modification gave a greater extension of circulatory half-life, but this was

**Figure 1** Tissue distribution over time of intact A5B7, with or without PEG modification, in the LS174T colonic xenograft model in nude mice. Results are expressed as percentage of injected antibody dose per $g^{-1}$ of tissue, and are the means of four mice. Vertical bars indicate s.d. ■, Intact A5B7; ▨, PEG–A5B7.

**Figure 2** Tissue distribution over time of $F(ab')_2$ A5B7, with or without PEG modification, in the LS174T colonic xenograft model in nude mice. Results are expressed as percentage of antibody dose per $g^{-1}$ of tissue, and are the means of four mice. Vertical bars indicate s.d. ■, $F(ab')_2$ A5B7; ▨, PEG-$F(ab')_2$ A5B7.

**Figure 3** Tissue distribution over time of Fab A5B7, with or without PEG modification, in the LS174T colonic xenograft model in nude mice. Results are expressed as percentage of antibody dose per $g^{-1}$ of tissue, and are the means of four mice. Vertical bars indicate s.d. ■, Fab A5B7; ▨, PEG–Fab A5B7.

accompanied by a further loss in antigen-binding activity, which had a detrimental effect on tumour localisation (data not shown).

PEG modification of intact A5B7 had little effect on biodistribution. Although the blood activity levels were slightly increased over the first 24 h when compared with the parent form (34.0% *vs* 25.3% at 3 h and 20.2% *vs* 14.0% at 24 h), this did not result in higher antibody levels in other normal tissues (Figure 1), and normal tissue to blood ratios remained very similar for the two groups throughout the experiment (data not shown). The tumour, however, showed a trend of decreased accumulation of intact A5B7 after PEG modification in spite of the raised blood levels, and inferior tumour to blood levels were found for all time points studied (Table I). Kitamura *et al.* (1991) found that the circulatory time of an intact anti-colon carcinoma antibody in a xenograft model was doubled after attachment of PEG, while tumour localisation was reduced. Levels in normal organs were either reduced (liver and spleen) or the same (lung and kidney) as found for the parent antibody. They suggest that reduced tumour levels may be caused by a blockade of transcapillary filtration owing to the higher molecular weight after PEG conjugation. It is thought that PEG-modified proteins have higher molecular weights than would be predicted by direct calculation of the number of PEG molecules attached (in the present case an additional 10 kDa per antibody molecule), probably because of hydration of the attached polymer (Knauf *et al.*, 1988).

Conjugation of PEG to the $F(ab')_2$ A5B7 approximately doubled the circulatory activity when compared with the unmodified fragments (Figure 2), resulting in higher accumulation in both tumour and normal tissues. This is in agreement with Kitamura *et al.* (1991), and is at least partly a reflection of reduced kidney filtration, although steric hindrance of non-specific binding and reduced proteolysis probably play a role. Renal clearance rate may be correlated with the molecular size of a protein, decreasing with increased size up to a threshold around $M_r$ 70,000, which is thought to be the permeability threshold of the kidney glomerular filtration system. The fact that the differential between circulatory clearance of PEG-modified and parent intact A5B7 ($M_r$ 150,000) was small in comparison with that found for the fragments is probably because the intact antibody was already above the size for renal filtration before polymer attachment. It is not clear how $F(ab')_2$ fragments, $M_r$ 100,000, are filtered by the kidney, and it is possible that a proportion is dissociated into the constituent Fab′ fragments. However, it is known that glomerular selectivity is based not only on molecular size, but also on shape and charge of the protein, and the attachment of PEG to the lysine side chains of the protein does appear to increase the negative charge of the molecule (Knauf *et al.*, 1988).

Parent Fab′ A5B7 had the lowest blood activity levels of the three forms of antibody studied (Figure 3), in agreement with rapid kidney clearance for a molecule that size ($M_r$ 50,000). After PEG modification, however, the size of the Fab′ molecule appeared to be raised above renal filtration limit, and the antibody behaved almost identically to the PEG-$F(ab')_2$ pharmacokinetically, with higher activity levels in tumour, blood and other normal tissues (Figures 2 and 3).

The increased circulatory half-life of antibody after PEG attachment, accompanied by higher tumour levels in the case of fragments, produced very similar tumour to blood ratios for all three forms of A5B7 at all time points studied, while the parent forms exhibited wide variations (Table I). The localisation index was always inferior to that of the unmodified antibody, and there was also little increase in the tumour to blood ratio with time. This was particularly true for $F(ab')_2$ A5B7, PEG modification of which reduced the tumour to blood ratio from 30:1 to 3.7:1 at 48 h and from 89.7 to 8.8:1 at 144 h after antibody delivery.

Although normal tissues also showed a higher accumulation of Fab′ and $F(ab')_2$ A5B7 after PEG modification, antibody specificity was improved because tissue to blood ratios were consistently around half those found for the parent fragments (data not shown). Kitamura *et al.* (1991) report lower levels of intact and $F(ab')_2$ anti-colon carcinoma antibody in normal tissues after PEG attachment, and suggest this may be caused by steric hindrance of non-specific antibody uptake. Other situations may also benefit, as we have found that PEG modification of a chimeric Fab′ fragment of A5B7 overcame the problem of high relative kidney accumulation, reducing the kidney to blood ratio from 20:1 to 1:1 a 24 h after administration (in preparation). Thus PEG conjugation enhanced tumour uptake while improving target specificity of the antibody.

One of the disadvantages of employing $F(ab')_2$ or Fab′ fragments rather than the intact antibody for RIT is reduced tumour localisation. After PEG modification the tumour levels were significantly raised, though they never reached those achieved by the intact A5B7 (Figures 1, 2 and 3). However, fragments do show deeper tumour penetration, thereby enhancing the range of cell kill at an early stage after administration when the intact antibody is still accumulated around the blood vessels (Boxer et al., 1994). Although we have still to determine whether PEG-modified fragments show altered tumour penetration, their use for therapy thus has the potential for increasing the tumour dose when compared with parent fragments, while possibly also improving the range of tumour cell kill over intact antibody. Circulating activity after administration of PEG-modified fragments was significantly lower than that of unmodified intact antibody, although a method of clearance such as a second antibody or an avidin-biotin system may be required to reduce potential damage to normal tissues if repeat dose therapy is used (Marshall et al., 1994). There appears to be little advantage in using the Fab' fragments in preference to the $F(ab')_2$ after PEG modification because, while normal tissues remain similar over time, the $F(ab')_2$ A5B7 shows higher tumour localisation (Figures 2 and 3; Table I).

A further advantage of PEG modification of proteins is the potential for reducing immunogenicity, possibly by shielding antigenic determinants with the immunologically inert polymer, and possibly by avoidance of reticuloendothelial cells (Francis et al., 1991). Kitamura et al. (1991) have shown that PEG attachment to an intact murine monoclonal antibody against human colon cancer reduces the HAMA response significantly when compared with the parent intact antibody. This is very important for RIT, as successful treatment requires repeated doses of antibody and an immune response would significantly reduce the effective radiation dose to the tumour (Ledermann et al., 1988).

In conclusion, we have demonstrated that PEG modification of an intact anti-CEA antibody has little effect on its subsequent biodistribution and clearance. In comparison, similar modification of its Fab' and $F(ab')_2$ fragments dramatically increased the plasma half-life, producing enhanced tumour accumulation and reduced normal tissue to blood ratios, although tumour to blood ratios were also reduced. The possibility of further reducing fragment immunogenicity by PEG attachment should therefore make them superior to either the parent forms or the intact antibody for all types of antibody-directed therapy. In addition, the feasibility of custom-designed PEG-antibody molecules for augmentation of biological activity, such as site-directed attachment and labile bonds, make this a useful tool for enhancing therapeutic potential.

This work was partly supported by the Cancer Research Campaign and the Ronald Raven Chair in Clinical Oncology Trust. We thank Drs G.E. Francis and C. Delgado for helpful discussion.

### References

BEGENT, R.H.J., LEDERMANN, J.A., GREEN, A.J., BAGSHAWE, K.D., RIGGS, S.J., SEARLE, F., KEEP, P.A., ADAM, T., DALE, R.G. & GLASER, M.G. (1989). Antibody distribution and dosimetry in patients receiving radiolabelled antibody therapy for colorectal cancer. *Br. J. Cancer*, **60**, 406-412.

BEGENT, R.H.J. & PEDLEY, R.B. (1990). Antibody targeted therapy in cancer: comparison of murine and clinical studies. *Cancer Treat. Rev.*, **17**, 373-378.

BOXER, G.M., ABASSI, A.M., PEDLEY, R.B. & BEGENT, R.H.J. (1994). Localisation of monoclonal antibodies reacting with different epitopes on carcinomembryonic antigen (CEA) – implication for targeted therapy. *Br. J. Cancer*, **69**, 307-314.

BUCHEGGER, F., PELEGRIN, A., DELALOYE, B., BISCHOF-DELALOYE, A. & MACH, J.-P. (1990). Iodine-131-labeled Mab (F(ab')2 fragments are more efficient and less toxic than intact anti-CEA antibodies in radioimmunotherapy of large human colon carcinoma grafted in nude mice. *J. Nucl. Med.*, **31**, 1035-1044.

DENARDO, S.J., DENARDO, G.L., O'GRADY, L.F., LEVY, M.B., MILLS, S.L., MACEY, D.J., MCGAHAN, J.P., MILLER, C.H. & EPSTEIN, A.L. (1988). Pilot studies of radioimmunotherapy of B cell lymphoma and leukemia using I-131 Lym-1 monoclonal antibodies. *Antibody Immunoconj. Radiophar.*, **1**, 17-33.

FRANCIS, G.E., DELGADO, C. & FISHER, D. (1991). PEG modified proteins. In *Pharmaceutical Biotechnology*, Vol. 3, *In vivo Pathways of Degradation and Strategies for Stabilisation*, Ahern, T.J. & Manning, M. (eds) pp. 235-263. Plenum Press: New York.

KITAMURA, K., TAKAHASHI, T.K, YAMAGUCHI, T., NOGUCHI, A., NOGUCHI, A., TAKASHINA, K-i., TSURUMI, H., INAGAKE, M., TOYOKUNI, T. & HAKOMORI, S.-I. (1991). Chemical engineering of the monoclonal antibody A7 by polyethylene glycol for targeting cancer therapy. *Cancer Res.*, **51**, 4310-4315.

KNAUF, M.J., BELL, D.P., HIRTZER, P., YOUNG, J.D. & KATRE, N.V. (1988). Relationship of effective molecular size to systemic clearance in rats of recombinant interleukin-2 chemically modified with water soluble polymers. *J. Biol. Chem.*, **263**, 15064-15070.

LEDERMANN, J.A., BEGENT, R.H.J., BAGSHAWE, K.D., RIGGS, S.J., SEARLE, F., GLASER, M.G., GREEN, A.J. & DALE, R.G. (1988). Repeated antitumour antibody therapy in man with suppression of the host response by cyclosporin A. *Br. J. Cancer*, **58**, 654-657.

MARSHALL, D., PEDLEY, R.B., BODEN, J.A., BODEN, R. & BEGENT, R.H.J. (1994). Clearance of circulating radio-antibodies using streptavidin or second antibodies in a xenograft model. *Br. J. Cancer*, **69**, 502-507.

NUCCI, M.L., SHORR, R. & ABUCHOWSKI, A. (1991). The therapeutic value of poly(ethylene glycol)-modified proteins. *Adv. Drug Delivery Rev.*, **6**, 133-151.

PEDLEY, R.B., BEGENT, R.H.J., BODEN, J.A., BODEN, R., ADAM, T. & BAGSHAWE, K.D. (1991). The effect of radiosensitizers on radioimmunotherapy, using $^{131}$I-labelled anti-CEA antibodies in a human colonic xenograft model. *Int. J. Cancer*, **47**, 597-602.

PEDLEY, R.B., BODEN, J.A., BODEN, R., DALE, R. & BEGENT, R.H.J. (1993). Comparative radioimmunotherapy using intact or $F(ab')_2$ fragments of $^{131}$I anti-CEA antibody in a colonic xenograft model. *Br. J. Cancer*, **68**, 69-73.

PEDLEY, R.B., BODEN, J., KEEP, P.A., HARWOOD, P.J., GREEN, A.J. & ROGERS, G.T. (1987). Relationship between size and uptake of radiolabelled anti-CEA in a colon tumour xenograft. *Eur. J. Nucl. Med.*, **13**, 197-202.

TOM, B.H., RUTZKY, L.H., JAKSTYS, M.M., OYASU, R., KAYE, C.I. & KAHAN, B.D. (1976). Human colonic adenocarcinoma cells. I. Establishment and description of a new cell line. *In Vitro*, **12**, 180-181.

TURNER, A., KING, D.J., FARNSWORTH, A.P.H., RHIND, S.K., PEDLEY, R.B., BODEN, J.A., BODEN, R., MILLICAN, T.A., BOYCE, B., BEALEY, N.R.A., EATON, M.A.W. & PARKER, D. (1994). Comparative biodistributions of $^{111}$-In macrocycle chimeric B72.3 antibody conjugates in tumour bearing mice. *Br. J. Cancer*, **70**, 35-41.